# Case report: Recurrent angioedema: Diagnosing the rare and the frequent

**DOI:** 10.3389/fmed.2022.1048480

**Published:** 2022-12-02

**Authors:** Thomas Buttgereit, Lauré M. Fijen, Carolina Vera, Karl-Christian Bergmann, Marcus Maurer, Markus Magerl

**Affiliations:** ^1^Institute of Allergology, Charité—Universitätsmedizin Berlin, Corporate Member of Freie Universität Berlin and Humboldt-Universität zu Berlin, Berlin, Germany; ^2^Fraunhofer Institute for Translational Medicine and Pharmacology ITMP, Allergology and Immunology, Berlin, Germany; ^3^Department of Vascular Medicine, Amsterdam Cardiovascular Sciences, Amsterdam University Medical Centers (UMC), University of Amsterdam, Amsterdam, Netherlands

**Keywords:** angioedema, recurrent, mast cell, omalizumab, normal C1INH, HAE

## Abstract

Hereditary angiodema with normal C1 inhibitor and unknown mutation (HAE-nC1INH-UNK), an exceedingly rare subtype of HAE, appears to be often diagnosed in patients who do not have this condition, but have mast cell-mediated angioedema. Here, we report two patients diagnosed with HAE-nC1INH-UNK by their physicians, who referred them to our center for treatment continuation with costly kallikrein-kinin-system targeted therapies. We describe how we established the correct diagnosis of recurrent mast cell-mediated angioedema after thorough investigation of both patients and initiated effective treatment with omalizumab. Also, we present and discuss the consensus criteria for diagnosing the very rare condition HAE-nC1INH in light of recent research and based on our own clinical experience. In conclusion, HAE-nC1INH-UNK should only be considered after more common differential diagnoses, i.e., mast cell-mediated angioedema, have thoroughly been investigated and ruled out. This approach reduces both the patients’ disease burden and healthcare costs and contributes to meaningful research.

## Introduction

**FIGURE 1:**
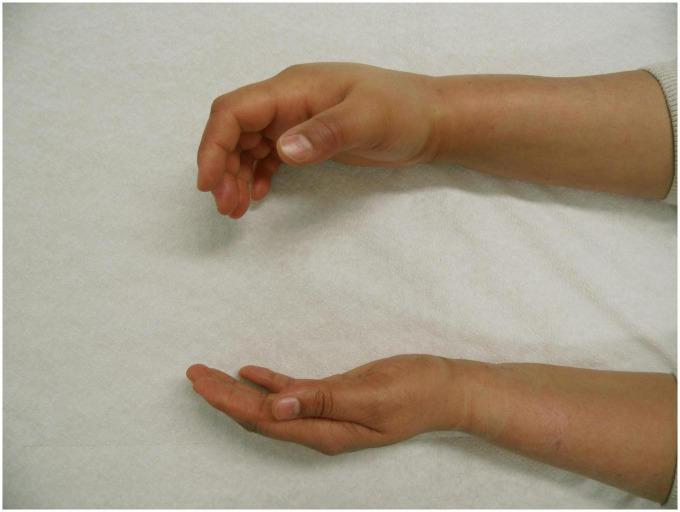
Angioedema of the right hand in a patient with hereditary angioedema due to C1 inhibitor (C1INH) deficiency (HAE-C1INH). Patient consent was obtained.

In 2000, two research groups independently described families with hereditary angioedema (HAE) in which C1 inhibitor (C1INH) levels were unremarkable (1, 2). Subsequently, this disease was named HAE with normal C1 inhibitor (HAE-nC1INH, formerly also called HAE type 3). Initially, very little was known about the pathomechanism of HAE-nC1INH, but the lack of response to antihistamines, cortisone and epinephrine argued against a mast cell-mediated mechanism. In 2006, the mystery seemed to be solved when a mutation in exon 9 of the *F12* gene was identified as the cause of HAE-nC1INH (3). Further research established that the development of angioedema (Figure 1) in these cases is due to uncontrolled formation of bradykinin. However, it soon became apparent that only a relatively small proportion of patients with HAE-nC1INH carried this disease-causing mutation, and the term HAE with normal C1 inhibitor of unknown cause (HAE-nC1INH-UNK) became common for patients with this phenotype but lacking a causative mutation. Although other causative mutations [*PLG* (plasminogen) (4), *ANGPT1* (angiopoietin 1) (5), *KNG1* (kininogen 1) (6), *MYOF* (myoferlin) (7), and *HS3ST6* (heparan sulfate 3-O-sulfotransferase 6) (8)] were identified in the past years, a molecular genetic confirmation of the very rare diagnosis of HAE-nC1INH still remains the exception.

In our experience from an Angioedema Center of Excellence and Reference (ACARE) (9), almost all patients with HAE-nC1INH are referred with the diagnosis of HAE-nC1INH-UNK based on their medical history, clinical symptoms, *ex juvantibus*, and the absence of laboratory findings indicative of other diseases. Since important and much more frequent differential diagnoses such as mast cell-mediated or ACE inhibitor-induced angioedema (AE-ACEI) cannot yet be diagnosed with the help of routine laboratory markers, but by using medical history, clinical symptoms, and therapeutic response, the diagnosis of HAE-nC1INH-UNK requires a cautious interpretation of the data and clinical experience. All too often, the supposedly well-defined diagnosis of HAE-nC1INH-UNK is chosen possibly to avoid, in the clinician’s opinion, less well-defined diagnoses such as mast cell-mediated angioedema or even the so-called diagnosis of idiopathic non-histaminergic angioedema. As a result, patients with a presumed or already established diagnosis of HAE-nC1INH-UNK are increasingly referred to our center for further diagnostic workup and treatment initiation, with high expectations in patients and referring physicians.

In this paper, we report two patients diagnosed with HAE-nC1INH-UNK by their physicians, who referred them for treatment continuation with kallikrein-kinin-system targeted therapies. Here, we describe how we established the correct diagnosis and initiated effective treatment. Also, we present the criteria for diagnosing the very rare condition HAE-nC1INH in light of recent research and based on our own experience.

## Case description

### Case 1

A male patient born in 1966 moved from the United States to Berlin, Germany in January 2015 and presented to our clinic for the first time in March of the same year. In the letter from his previously treating physicians, the patient was diagnosed with HAE-nC1INH and we were asked to continue treatment with plasma-derived C1INH concentrate 1,000 units intravenously twice a week and icatibant, ecallantide, or epinephrine injector as needed in emergent cases. In the medical history taken at our ACARE, the patient reported recurrent angioedema since about the age of 20 years. Swellings had initially occurred on the tongue and face, and later the patient reported about swellings on the chest and painful abdominal symptoms, too. When asked in more detail, he had also experienced wheals from time to time. Since prophylactic treatment with 2nd generation H1-antihistamines up to the four-fold dose and corticosteroids had been insufficiently effective (at times the patient had to visit the emergency department several times a month), the diagnosis was changed from “recurrent idiopathic angioedema” to HAE-nC1INH some years before he was transferred. Mutational analysis was not performed, family history was negative, except a paternal uncle reported a once in his lifetime swelling. After the establishment of the diagnosis HAE-nC1INH, the patient received danazol at a dose of 200 mg; and when C1INH concentrate became available in the United States, the patient was switched to long-term prophylaxis with plasma-derived C1INH concentrate as described above. Other diagnoses according to physician reports included attention deficit hyperactivity disorder, gastroesophageal reflux, bipolar disorder, status post pulmonary embolism in March 2008, asthma, and vocal cord dysfunction. For treatment of these disorders, the patient was taking amphetamine dextroamphetamine, cholecalciferol, ciclesonide, fish oil, fluticasone, levocetirizine, methocarbamol, vitamin tablets, omeprazole, trazodone, and valaciclovir.

As the patient reported that since moving to Germany the swelling attacks had decreased significantly, he used treatment in the last months only as needed. Laboratory parameters obtained at his initial presentation confirmed normal C1INH activity and concentration (128%; 0.26 g/L), normal tryptase, and moderately elevated total IgE (5.79 μg/L; 139 KU/L). Although the patient was able to self-administer C1INH or icatibant, he occasionally came to the clinic for treatment and monitoring. On two of these occasions, our ACARE physicians observed anxiety and agitation, with shortness of breath and a marked expiratory stridor. After injection of icatibant, improvement occurred after about 30 min, just as after injection of C1INH concentrate. The patient asserted that these were the exact symptoms of his HAE disease. After each treatment, the patient could be discharged about 1 h after treatment. At the next regular appointment, we diagnosed the patient with both non-allergic bronchial asthma and mast cell-mediated angioedema in the setting of chronic spontaneous urticaria (CSU) refractory to antihistamines. Subsequently, we initiated treatment with omalizumab 300 mg subcutaneously every 4 weeks in June 2015. Regarding his non-allergic bronchial asthma, the patient received budesonide, formoterol (both once daily) and salbutamol (as required). Angioedema occurrence seized immediately, asthmatic symptoms markedly improved after a few weeks and almost completely disappeared in the further course. C1INH concentrate and icatibant no longer had to be used. Since the start of omalizumab treatment, the patient had no more angioedema attacks, except for once, when he attempted to prolong the injection interval of omalizumab. In August 2022, his disease control was complete as assessed by use of the angioedema control test (AECT, 16 points). Nevertheless, the impairment of quality of life was still considerable as assessed by use of the angioedema quality of life questionnaire (AE-QoL 53 points: Functioning: 0 Points, Fatigue/Mood: 65 Points, Fears/Shame: 91 Points, Nutrition: 12 Points), possibly overlaid by feelings of fear and impairment from his history of recurrent angioedema and/or comorbid bipolar disorder.

### Case 2

A male patient born in 1958 moved to Berlin in September 2021 from another European country for almost 1 year for professional reasons. His referring physician, who had diagnosed HAE-nC1INH, reached out to us and asked us to continue treatment with lanadelumab 300 mg every 14 days, which had improved the patient’s condition. The medical history taken at our ACARE confirmed that the patient had recurrent angioedema, which had started at the age of 56 years, with swellings have mainly occurring on the face, tongue, and genitals. Furthermore, the patient stated that swellings usually developed in the early morning hours, usually initially hemifacial. Prophylactical treatment with double-dose 2nd generation H1-antihistamines (cetirizine, bilastine) did not control the disease; and corticosteroids were never used. After the patient swelling attacks responded to icatibant several times and the attack rate increased significantly in the further course, he was diagnosed with HAE-nC1INH, and long-term prophylaxis with lanadelumab, 300 mg subcutaneously every 2 weeks, was initiated. With this, the patient observed clear improvement, and on-demand therapy with icatibant was no longer necessary. However, the patient was not completely symptom-free. When there was an unintended interval extension of lanadelumab for several weeks shortly after his relocation to Germany due to unresolved insurance issues, the symptoms worsened and regressed relatively slowly after re-initiation of lanadelumab. At his visit at our ACARE in February 2022, the patient scored four points (meaning uncontrolled disease) in AECT and 37 points in the AE-QoL. The patient’s history also revealed that he had experienced wheals from time to time. In his family history, only his maternal grandmother had a history of infrequent ocular swellings. Laboratory parameters obtained at our ACARE confirmed normal C1INH activity and concentration (126%; 0.3 g/L), normal tryptase and elevated total IgE (7.05 μg/L; 320 kU/L). Molecular genetic testing for the genes ADGRE2, ANGPT1, CPN1, F12, KNG1, NLRP3, PLCG2, PLG, SERPING1, SPINK5, TNFAIP3 was negative with respect to mutations known to cause HAE. As an incidental finding, a genetic variant in PLCG2, c.656 A > G p.(Asp219Gly) of unclear significance was identified, which is associated with familial autoinflammatory cold syndrome-3, for which, however, there was no history or clinical evidence in the patient. Based on the patients’ medical history, clinical features, and his insufficient response to lanadelumab, we suspected mast cell-mediated recurrent angioedema due to CSU and initiated treatment with omalizumab in the approved dose; lanadelumab was discontinued. Already after the first injection with omalizumab, the patient was completely symptom-free, and he scored 15 points (well-controlled disease) in the AECT in June 2022. In September 2022, his disease control was complete (AECT 16 points), and the impairment of quality of life was minimal (AE-QoL 3 points).

## Discussion

These two patient cases demonstrate that, as often reported, not only patients with HAE are misdiagnosed, i.e., with mast cell-mediated angioedema, and have a diagnostic delay, but that misdiagnosis of HAE can also happen in the other direction. In particular, HAE-nC1INH-UNK, an exceedingly rare subtype of HAE, appears to be often diagnosed in patients who do not have this condition, but have mast cell-mediated angioedema. In both cases presented here, the diagnosis of HAE-nC1INH was made despite features pointing to mast cell-mediated angioedema and without genetic testing. This led to significant consequences for the patients and caused high costs for the healthcare system. The correct diagnosis of HAE-nC1INH is challenging and there are several things that need to be considered, which we will discuss in more detail here.

**FIGURE 2:**
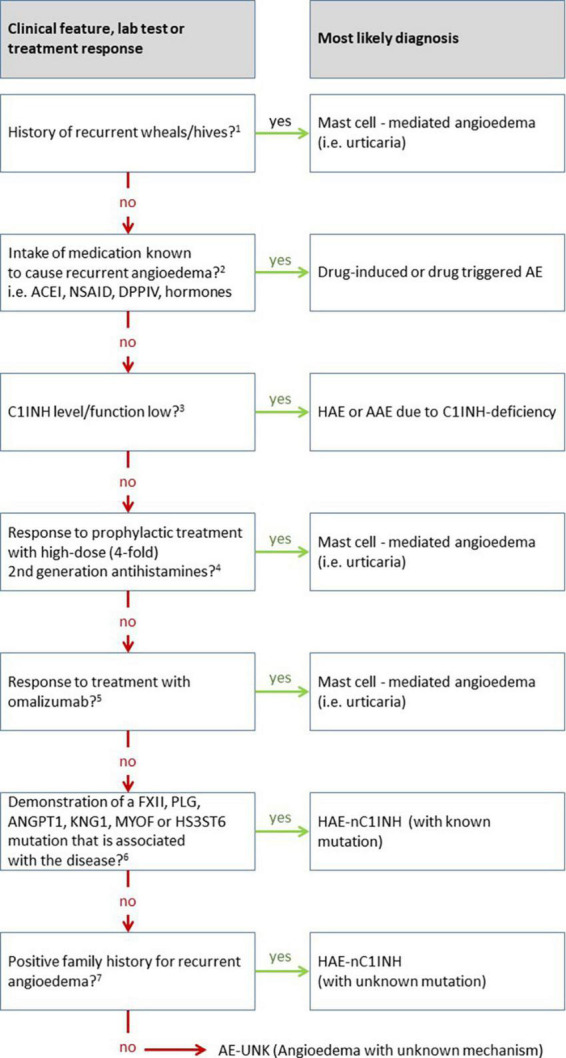
Overview of the differential diagnosis of hereditary angioedema (HAE) with normal C1 inhibitor (HAE-nC1INH). This diagram is adapted from the work of Zuraw et al. (11), updated based on own experience, newly approved therapeutic options in the meantime, and additional mutations discovered since then. ^1^The recurrent occurrence of short-lived pruritic wheals and angioedema (AE) in recent patient’s history is most suggestive of the presence of mast cell-mediated angioedema, i.e., chronic urticaria. However, the presence of wheals does not completely exclude the presence of another disease other like HAE, since urticaria, a very common disease, may also occur in association with HAE (approximately 1% of cases). ^2^Medications can cause various forms of angioedema or trigger underlying diseases causing angioedema. For example, ACE inhibitors (ACEI) can cause swelling in otherwise healthy individuals but can also very reliably trigger swelling in patients with C1INH inhibitor deficiency. On the other hand, non-steroidal anti-inflammatory drugs (NSAID) can trigger swelling in otherwise healthy people, but also in many cases trigger angioedema flare-ups in patients with urticaria. ^3^Angioedema due to C1INH inhibitor (C1INH) deficiency typically results in markedly decreased values for C1INH concentration and/or function. Borderline depressed results are not likely to attribute angioedema to C1INH deficiency. ^4^Verification of the efficacy of antihistamines can be meaningfully evaluated by the administration of prophylaxis alone. The response of an as-needed therapy can rarely be measured validly in individual cases *in praxi*. The duration of prophylaxis must be adapted to the frequency of angioedema, too short administration for false negative results. The dose should and can be increased to four times the usual daily dose for modern non-sedating 2nd generation antihistamines, e.g., up to 20 mg levocetirizine or desloratadine; 40 mg cetirizine, loratadine, rupatadine, or ebastine; 80 mg bilastine. It is recommended to use a lower dose (single or double the usual daily dose) before the maximum dose is applied, as this may also be sufficient in some cases. Non-efficacy of antihistamines does not justify the term non-histaminergic angioedema, since it is known that antihistamines are not sufficiently effective in more than 50% of cases of chronic spontaneous urticaria (CSU). ^5^A significantly higher responder rate compared to antihistamines is seen with the use of omalizumab, both for wheals and angioedema. The response of omalizumab to angioedema is so reliable that in the absence of efficacy of omalizumab, the involvement of mast cells in the disease may be doubted. The use of omalizumab is a key step in the diagnosis of hereditary angioedema with normal C1 inhibitor when no underlying mutation for HAE-nC1INH is found. ^6^Molecular genetic workup should ideally include all known mutations, or at least the commonly described ones involving factor 12 (FXII), plasminogen (PLG), or kininogen (KNG). In all likelihood, the number of causative mutations to be tested will continue to increase over the next several years. ^7^If no causative mutation can be found in the molecular genetic analysis, the family history becomes of crucial importance. In this case, the family history must be clear and verifiable with regard to angioedema. For a definite diagnosis, several family members should be affected in more than one generation. The assumption that a single patient without family history suffers from angioedema as a result of a *de novo* mutation of an unknown gene is inadmissible.

Hereditary angioedema due to C1INH deficiency (HAE-C1INH) is an orphan disease, with an estimated prevalence of 1:50.000 (10). The prevalence of HAE-nC1INH, and even more specifically of HAE-nC1INH-UNK, is much lower, making it extremely rare. Meanwhile, other diseases that present with recurrent angioedema, such as mast cell-mediated angioedema and drug-induced angioedema, are much more common. Thus, epidemiologically, HAE-nC1INH-UNK should not be presumed until more plausible explanations for recurrent angioedema are ruled out (Figure 2). In 2012, an international expert panel developed consensus criteria for the diagnosis of HAE-nC1INH (11). First, HAE-nC1INH requires a history of recurrent angioedema in the absence of concomitant hives/wheals or concomitant use of a medication known to cause angioedema. Second, HAE-nC1INH should only be diagnosed with documented normal or near normal C4 levels, C1INH levels, and C1INH function. Third, the following features need to be present: (1) demonstration of a *F12* mutation that is associated with the disease, or (2) a positive family history of angioedema, and (3) documented evidence of lack of efficacy of continued high-dose antihistamine therapy (cetirizine at 40 mg/day or equivalent, for at least 1 month and an interval expected to be associated with three or more attacks of angioedema). We consider the absence of concomitant wheals/hives as particularly relevant for diagnosing HAE-nC1INH. It is important to note that “concomitant,” in this case, means the occurrence of wheals (hives) at any time during the course of the disease, but not necessarily at the same time as the angioedema is present. Wheals, in CSU patients who experience wheals and angioedema, oftentimes present in the absence of angioedema and vice versa. Approximately 20% of the general population experiences a single occurrence of wheals in their lifetime (12), unrelated to mast cell- mediated angioedema. On the other hand, up to 10% of many patients with mast cell-mediated angioedema do never experience wheals (13). Therefore, additional criteria as stated by Zuraw et al. (11) must also be met before HAE-nC1INH is diagnosed.

When the first two criteria are met and mutations in the *FXII* (3), *PLG* (4), *ANGPT1* (5), *KNG1* (8), *MYOF* (7), and *HS3ST6* (8) genes associated with HAE are ruled out, a positive family history is needed for the diagnosis of HAE-nC1INH. Importantly, the family history must be truly positive, with at least one, but preferably several family members, and ideally multiple generations affected. Other family members affected need to have experienced signs and symptoms indicative of HAE and responses to medication that are compatible with HAE. To put it bluntly, rumors that the grandmother once had swollen legs do not suffice. In the absence of a crystal-clear family history and a causative HAE mutation, the patient should be considered to have non-hereditary angioedema rather than HAE-nC1INH. In addition to a true positive family history, HAE-nC1INH-UNK may only be diagnosed if treatment with high-dose 2nd generation antihistamines has been shown to be ineffective. Here, several things are important to consider. First, even in *bona fide* mast cell-mediated angioedema, high-dose antihistamines have been shown to be ineffective in most patients, whereas, the vast majority patients with antihistamine-refractory angioedema respond to omalizumab (14–16). In other words, non-response to high-dose antihistamines does not necessarily indicate a non-mast cell mediated cause of recurrent angioedema. Therefore, we propose using ineffective treatment with omalizumab for at least 6 months as a criterion for diagnosing HAE-nC1INH. Although non-response to omalizumab does not rule out mast cell-mediated angioedema either, it does make it very unlikely. Of note, the efficacy of on demand use of antihistaminergic drugs to differentiate between mast cell-mediated and bradykinin-mediated recurrent angioedema is of little value. Reasons for this include the lack of validated, objective parameters to measure the efficacy of acute treatment of angioedema (17, 18) and the often-unrealistic expectations of both patients and physicians regarding the efficacy of these acute treatments. On demand antihistamine medication primarily prevents further progression of the swelling rather than promoting its regression. Moreover, the response to on demand treatment with antihistamines is dependent on the localization and severity of angioedema, the time between the start of swelling symptoms and the administration of the medication, and the dosage and route administration of the medication used. Given the enormous heterogeneity of angioedema symptoms and on demand use of antihistamines, it is downright impossible to accurately measure the responsivity to antihistamine treatment used at a single time. In contrast, the efficacy of angioedema prophylaxis can readily be measured with validated instruments, i.e., by using patient reported outcome measures (PROM) for disease activity [e.g., Angioedema Activity Score, AAS (19)], impact [AE-QoL (20)] and control [AECT (21)]. Hence, it is much more appropriate to evaluate the efficacy of prophylactic antihistaminergic therapy as opposed to on demand antihistaminergic medication for the treatment of an angioedema attack when assessing efficacy of antihistamines. This is recommended to be done with a high-dose antihistamine treatment (cetirizine 40 mg/day or equivalent) for 1 month or the duration of an interval in which at least three angioedema attacks are expected to happen in individual patients, whichever lasts longer (11).

Interestingly, both patients with recurrent mast cell-mediated angioedema benefitted from medication that acts very specifically on the kinin-kallikrein system, namely icatibant, C1INH concentrate, and lanadelumab. Even though the expectations of patients and physicians when using such drugs are very high and a pronounced placebo effect can be assumed, the described effects appear to go far beyond this. In recent years, there has been increasing evidence that there are numerous functional cross-links between mast cells and the kinin-kallikrein system that challenge the classification of angioedema as either exclusively bradykinin-mediated or exclusively mast cell-mediated (22). These links between the two systems may explain, at least in part, the partial response of both patients. Moreover, the bradykinin B2 receptor antagonist icatibant inhibited carrageenan-induced angioedema in rats, which involves mast cells and histamine (23, 24). More recently, a clinical trial demonstrated efficacy for C1INH administration in human asthma (25), which is also, in part, mediated by mast cells and histamine. These findings and the partial response of our patients suggest that treatments for bradykinin-mediated angioedema including HAE may have benefit in mast cell-mediated angioedema, but this has not yet been investigated in controlled studies.

Misdiagnosis of HAE-nC1INH-UNK can have negative consequences in many respects, and the experience of our two patients underlines this. The diagnostic delay caused by misdiagnosing HAE-nC1INH-UNK prolongs disease burden, as patients will not be free of angioedema attacks. Recurrent angioedema attacks have been shown to greatly impact the quality of many aspects of patients’ lives, not only physically, but also because of their effects on mental health, reproductive choices, social relationships, productivity, and work performance (26–30). Furthermore, on demand and prophylactic treatments for HAE-nC1INH (25) are very costly. When these medications are prescribed but have limited or no efficacy in patients incorrectly diagnosed with HAE-C1INH-UNK, healthcare costs unnecessarily increase. Last, but not least, incorrect diagnosis also impacts meaningful research. When the diagnostic criteria for HAE-C1INH are not met, outcomes of research investigating these patients are unreliable and thus unimplementable.

In conclusion, HAE-nC1INH-UNK should only be considered after more common differential diagnoses, i.e., mast cell-mediated angioedema, have thoroughly been investigated and ruled out. When HAE-nC1INH-UNK is considered as the explanation for recurrent angioedema, the consensus criteria, updated for mutations and modern treatments discovered after the consensus meeting took place, should be observed to the letter.

## Data availability statement

The original contributions presented in this study are included in this article/supplementary material, further inquiries can be directed to the corresponding authors.

## Ethics statement

Ethical review and approval was not required for the study on human participants in accordance with the local legislation and institutional requirements. The patients/participants provided their written informed consent for the publication of this case report.

## Author contributions

MMg, TB, and LF contributed to the conception and design of the study and wrote the first draft of the manuscript. K-CB assisted in the diagnosis and therapy of patients. All authors contributed to the manuscript revision, read, and approved the submitted version.
